# A comparison of the efficacy and safety of Chinese patent medicine combined with Western medicine for *Helicobacter pylori*-related gastric ulcer: A systematic review and network meta-analysis

**DOI:** 10.1097/MD.0000000000041137

**Published:** 2025-02-07

**Authors:** Meiqi Zhong, Qifang Sun, Baoping Ren, Chang Yu, Shunhua Zhou, Qing Gao, Xiaojuan Wang, Chengzhi Yuan, Jing Lu, Qinghua Peng, Meiyan Zeng, Houpan Song

**Affiliations:** a Hunan Provincial Key Laboratory of Traditional Chinese Medicine Diagnostics, Hunan University of Chinese Medicine, Changsha, Hunan, China; b School of Traditional Chinese Medicine, Hunan University of Chinese Medicine, Changsha, Hunan, China; c School of Medicine, Hunan University of Chinese Medicine, Changsha, Hunan, China

**Keywords:** Chinese patent medicine, combined therapy, gastric ulcer, *Helicobacter pylori*, network meta-analysis

## Abstract

**Background:**

The aim of this network meta-analysis (NMA) was to compare the effectiveness and safety of different Chinese patent medicines (CPMs) combined with Western medicines (WMs) regimen versus WMs alone in the treatment of *Helicobacter pylori*-related gastric ulcer (GU).

**Methods:**

A comprehensive search was conducted on databases from their inception to May 31, 2023, to identify all randomized controlled trials (RCTs) that investigated the efficacy of CPMs in combination with conventional WMs in the treatment of patients with *H pylori*-related GU. Using Cochrane risk of bias assessment tool, we evaluated the methodological quality of RCTs. R version 4.2.3 and Stata version 15.1 software were cross-merged to conduct pairwise NMA.

**Results:**

A total of 35 studies involving 4667 patients and 11 CPMs were identified. Eleven CPMs were analyzed, including Pingwei Capsule (PWC), Kangfuxin Solution (KFXS), Shugan Jieyu Capsule (SGJYC), Weisu Granule (WSG), Qiwei Weitong Capsule (QWWTC), Beiling Weitong Granule (BLWTG), Anweiyang Capsule (AWYC), Jinghua Weikang Capsule (JHWKC), Weifuchun Tablet (WFCT), Wenweishu Capsule (WWSC), and Weidean Capsule (WDAC). Results showed that the combination of CPM and WM was more effective relative to the WM regimen alone. NMA revealed that WWSC combined with the WM yielded superior results in enhancing clinical outcomes and mitigating GU recurrence rates. PWC combined with the WM showed the best performance in improving the *H pylori* eradication rate. WFCT combined with the WM had the most optimal performance in controlling gastrin (GAS) and motilin (MTL) levels. KFXS combined with the WM showed the best results in terms of reducing the incidence of adverse events.

**Conclusion:**

Our NMA findings indicate that the combination of WWSC, PWC, WFCT, and KFXS with WM may be more effective and advantageous outcomes compared to other CPMs. Due to the limitations of this study, future research should employ larger sample sizes and multicenter RCTs to conduct real-world clinical studies.

## 1. Introduction

Gastric ulcer (GU) is a prevalent disease in the upper digestive tract, with a global incidence of 10%.^[1]^ Its occurrence is attributed to multifactorial etiologies, including *Helicobacter pylori* infection, smoking, nonsteroidal anti-inflammatory drug intake, and excessive alcohol consumption.^[2]^ Studies have shown that *H pylori* infection is the primary etiology of GU and is widespread among approximately 50% of the global populace.^[3]^ The common manifestations of *H pylori*-infected GU include abdominal pain, distension, belching, acid reflux, and exacerbation of pain post-prandially, with a melioration preceding meals.^[4]^ Several drugs are available for treating *H pylori*-related GU, including antibiotics, antacids, anticholinergics, proton pump inhibitors, and H2-receptor antagonists.^[5]^ Although conventional medical approaches may alleviate some of the aforementioned symptoms, the high recurrence rate of GU, prolonged disease duration, and incomplete eradication of *H pylori* often limit the efficacy of such interventions.^[6]^

The progressive advancement in experimental and clinical research of traditional Chinese medicine (TCM) has resulted in its recognition as a supplementary therapy alongside Western medicine (WM) in the management of GU clinical conditions. The efficacy and safety of TCM have been increasingly substantiated, hence an essential component in the medication regimen for these conditions. In recent years, randomized controlled trials (RCTs) combining Chinese patent medicines (CPMs) with Western medicines (WMs) have been the predominant approaches in clinical research on the treatment of *H pylori*-related GU. However, comparative studies examining the efficacy and safety of various CPMs in treating *H pylori*-related GU are significantly limited.^[7]^ Due to the challenging nature of managing *H pylori*-related GU, there is a need within the global gastroenterology community to ascertain the efficacy of various types of CPM when combined with WM therapy in alleviating GU symptoms. Additionally, it is important to establish which specific CPM, in conjunction with WM, yields the highest efficacy. However, the existing literature lacks a comprehensive systematic review or network meta-analysis (NMA) investigating the use of CPMs combined with Western medicines (WMs) for the treatment of GU due to *H pylori* infection. Unlike pairwise meta-analyses, NMA can summarize direct and indirect evidence and compare the relative efficacy of multiple treatments, thereby promoting better clinical decision-making. NMA can simultaneously compare the therapeutic effect differences among multiple interventions in the evidence body and rank them based on the effect size.^[8]^ Herein, a systematic review and NMA were performed to compare the efficacy and safety of various combinations of CPMs and WMs in treating GU caused by *H pylori* infection. This work aims to rank these treatments and provide evidence-based support for the clinical management of *H pylori*-related GU.

## 2. Methods

The research strictly adhered to the PRISMA statement guidelines for conducting systematic reviews and network meta-analyses. The PROSPERO registration ID is CRD42023474100. As this study does not involve human or animal testing or case reports or a series of cases, ethical approval from the Ethics Committee and Institutional Review Board was not required.

### 2.1. Retrieval strategy

Two authors independently conducted literature searches, used selection criteria, extracted data, assessed quality, and performed statistical analyses. Both authors double-checked their work. Electronic databases, including China National Knowledge Infrastructure (CNKI), PubMed, Wanfang, China Science and Technology Journal Database (VIP), and Embase were searched. The following keywords were used to search databases from inception to May 31, 2023, for correlative RCTs: “gastric ulcer,” “gastrohelcosis,” “gastrohelcoma,” “*Helicobacter pylori* infection,” “traditional Chinese medicine,” “Chinese patent medicine,” “Western medicine,” “triple therapy,” “quadruple therapy,” “randomized controlled trial,” and “RCT.”

### 2.2. Eligibility criteria

Inclusion criteria were study design: RCTs of CPM combined with WM in the treatment of GU patients infected with *H pylori*. It was determined that each patient met the diagnostic criteria for *H pylori*-positive GU. GU was diagnosed by endoscopy and pathology, and ^14^C-UBT was positive. GU belonged to the active ulcer stage. The patient had a high degree of cooperation and good compliance. Interventions: The control group received triple or quadruple therapy and other Western drugs. The experimental group was treated with CPMs based on the control group. Outcomes: The primary outcome included the rate of clinical efficacy and *H pylori* eradication, which were evaluated according to diagnosis and treatment of peptic ulcer in TCM efficacy. The secondary outcomes included GAS levels and MTL levels. Safety outcomes included the rate of adverse events and GU recurrence.

Exclusion criteria were non-RCTs (*r*eviews or meta-analyses, experimental research, retrospective studies, case reports, or duplicated reports). Patients with malignant tumors of the digestive tract and an operation history of the upper digestive tract. Patients with other diseases (heart, kidney, liver, and other serious functional abnormalities, mental illness, and other important organ problems). Pregnant or lactating women. Incomplete data or inaccessible full text.

### 2.3. Data collection

Data extraction and collation were performed independently by 2 investigators using a standardized Microsoft Excel spreadsheet. Conflicts were resolved by consensus between the 2 researchers or, if necessary, by consulting with another investigator. The data extracted included: the name of the main author, the publication year, and the characteristics of participants, including gender, average age, and number of participants. Several intervention measures were incorporated into the experimental and control groups, including drugs, dosage, frequency, and duration of treatment.

### 2.4. Risk of bias assessment

The assessment of study quality was conducted using the Cochrane risk-of-bias tool, which encompasses various evaluation components including the generation of random sequences, concealment of allocations, blinding of subjects and result assessors, integrity of data, and selective reporting. The aforementioned quality evaluation was categorized into 3 grades: “unclear risk,” “low risk,” and “high risk.” Two researchers independently assessed the quality, and any disagreements were resolved through discussions involving a third party.

### 2.5. Statistical analysis

An assessment instrument using bias risk was adopted to assess the methodological rigor of implementation literature, whereas bias risk plots were generated using RevMan 5.3 software. Standard pairwise and Bayesian NMA were conducted using Stata version 15.1 and R version 4.2.3 software. Odds ratios (ORs) with 95% confidence intervals (CIs) were computed to determine the intervention effect of combining CPM with WM for dichotomous data. Mean change (the disparity between pre- and post-intervention outcomes), mean, and standard deviation were analyzed for continuous data samples.

The effect size refers to mean differences with 95% CIs for continuous data. Based on the theory of likelihood functions and a few previous assumptions, Markov Chain Monte Carlo (MCMC) simulations were performed using Bayesian inference with R 4.2.3 software. Notably, 500,000 iterations and 20,000 annealings were set to investigate the posterior distributions of the interrogated nodes.^[9,10]^ To assess statistical heterogeneity among the studies, we employed *I*^2^ statistics within the R software, which quantifies the proportion of total variation in effect sizes between studies that can be attributed to heterogeneity rather than sampling error. A heterogeneity level of 25% was deemed low, 50% moderate, and 75% substantial. A random effect model was utilized if significant heterogeneity was observed; otherwise, we used a fixed effect model. Furthermore, we generated comparative adjusted funnel plots using Stata version 15.1 to examine publication bias.^[11]^

Sensitivity analyses were performed to evaluate the resilience of the findings and address any potential heterogeneity. Based on Stata network package, we constructed the network relationship diagram, network evidence diagram, funnel diagram, and surface under the cumulative ranking curve (SUCRA) ranking diagram. Plots of the forest were generated with the forest plot package in R. For each outcome, indicators of SUCRA were used to determine the stand or fall of interventions for sorting. A SUCRA value, representing the percentage of the area under the cumulative rank probability curve was used to assess treatment efficacy. This value ranged from 0 to 100%, with higher values indicating greater efficacy.^[12]^ Cluster analysis was conducted to evaluate the selected CPMs + WMs within each outcome, including the clinical efficacy rate, *H pylori* eradication rate, GAS and MTL levels, GU recurrence rate, and incidence of adverse events.

## 3. Results

### 3.1. Literature search and screening

**Figure 1.**
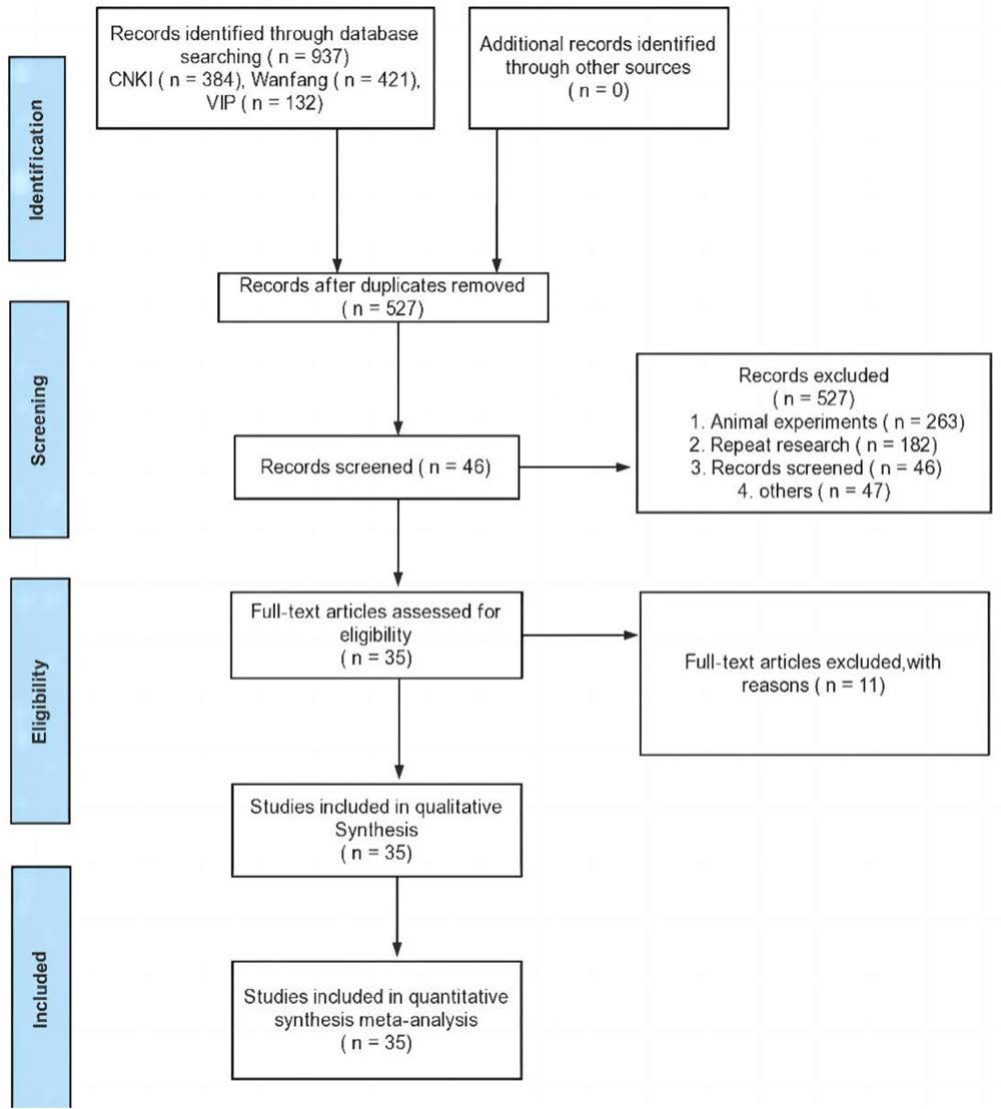
Flow chart showing selection process of studies for the final inclusion.

Figure 1 shows the literature search and selection strategy. A total of 1874 studies were identified from research literature-related databases. In total, 46 articles were retained after removing 527 duplicates and 1301 studies unrelated to the theme, including reviews, experimental research, retrospective studies, case reports, and protocols. Based on our criteria, 11 literature sources lacking control studies or pertinent outcome indicators were further removed after a thorough evaluation of the complete texts. Consequently, the remaining 35 literature sources were deemed suitable for inclusion in the study.

### 3.2. Study characteristics

In total, 4667 participants were recruited into the study in 35 pairwise comparisons, with the majority of studies originating from China. The RCTs were conducted on patients diagnosed with *H pylori*-related GU and admitted to various hospitals. The experimental group comprised individuals assigned to 11 different CPMs, including PWC (n = 3), KFXS (n = 4), SGJYC (n = 4), WSG (n = 3), QWWTC (n = 2), BLWTG (n = 2), AWYC (n = 3), JHWKC (n = 4), WFCT (n = 4), WWSC (n = 3), and WDAC (n = 3). Conversely, the control group solely received WM, whereas the experimental group received CPM and WM combination. Table 1 shows comprehensive study characteristics, including patient gender and age, treatment duration, interventions, and outcomes.

**Table 1:** Characteristics of the included studies.

Study ID	N (E/C)	Sex (M/F)	Age (yr)	Therapy of treatment group	Therapy of control group	Course	Outcomes
Hu CY (2018)^[6]^	43/43	E: 25/18 C: 27/16	E: 39.72 ± 5.43 C: 40.13 ± 6.25	AWYC + WM	WM: quadruple therapy	4 wk	①②⑥
Zhang J (2014)^[13]^	40/40	E: 24/16 C: 26/14	E: 37.07 ± 2.13 C: 36.72 ± 2.43	AWYC + WM	WM: triple therapy	4 wk	③④⑥
Pan LQ (2016)^[14]^	37/37	E: 21/16 C: 18/19	E: 42.2 ± 1.8 C: 45.5 ± 1.5	PWC + WM	WM: proton pump inhibitor	4 wk	①⑥
Yu AL (2015)^[15]^	500/500	E: 312/188 C: 306/194	E: 45.6 ± 2.6 C: 45.3 ± 2.4	PWC + WM	WM: proton pump inhibitor	4 wk	①②
Wang QN (2019)^[16]^	80/80	E: 46/34 C: 45/35	E: 45.82 ± 9.37 C: 45.54 ± 9.22	KFXS + WM	WM: quadruple therapy	1 mo	①
Guo HC (2022)^[17]^	36/36	E: 21/15 C: 22/14	E: 33.24 ± 6.27 C: 32.54 ± 6.27	KFXS + WM	WM: quadruple therapy	E: 2 wk C: 4 wk	①②⑤⑥
Tan DS (2022))^[18]^	55/55	/	/	KFXS + WM	WM: quadruple therapy	6 wk	①②⑤⑥
Zhang HY (2017)^[19]^	124/124	E: 74/50 C: 75/49	E: 46.09 ± 3.17 C: 47.30 ± 3.04	KFXS + WM	WM: triple therapy	40 d	①②⑤
Yang L (2021)^[20]^	46/46	E: 29/17 C: 28/18	E: 40.71 ± 2.12 C: 40.65 ± 2.13	SGJYC + WM	WM: quadruple therapy	15 d	①②
Zhou M (2015)^[21]^	45/44	E: 26/19 C: 26/18	E: 39.1 ± 9.7 C: 40.2 ± 9.8	SGJYC + WM	WM: quadruple therapy	6 wk	①②③④⑥
Zhang Y (2018)^[22]^	51/51	E: 30/21 C: 30/21	E: 38.9 ± 11.9 C: 38.5 ± 12.1	SGJYC + WM	WM: quadruple therapy	6 wk	①②③④⑥
Tan QJ (2022)^[23]^	34/34	E: 15/19 C: 18/16	E: 62.67 ± 2.42 C: 62.58 ± 2.37	WSG + WM	WM: quadruple therapy	30 d	①②⑤⑥
Qin YH (2021)^[24]^	36/36	E: 21/15 C: 19/17	E: 47.82 ± 13.54 C: 46.95 ± 14.68	WSG + WM	WM: quadruple therapy	E: 6 wk C: 4 wk	①⑤
Yu JJ (2020)^[25]^	43/43	/	/	WSG + WM	WM: quadruple therapy	4 wk	①②⑤
Wang XN (2019)^[26]^	52/51	E: 31/21 C: 28/23	E: 46.52 ± 1.56 C: 47.34 ± 11.08	QWWTC + WM	WM: quadruple therapy	6 wk	①②
Chen T (2019)^[27]^	42/42	E: 19/23 C: 20/22	E: 41.46 ± 2.25 C: 41.22 ± 2.14	QWWTC + WM	WM: quadruple therapy	6 wk	①⑤⑥
Ji ZH (2022)^[28]^	48/48	E: 32/16 C: 31/17	E: 58.63 ± 5.34 C: 58.32 ± 5.28	BLWTG + WM	WM: quadruple therapy	4 wk	①②③④⑥
Yu L (2021)^[29]^	34/34	E: 18/16 C: 20/14	E: 25-65 C: 23-65	BLWTG + WM	WM: quadruple therapy	6 wk	①②
Zhang CY (2021)^[30]^	42/42	E: 24/18 C: 22/20	E: 48.46 ± 10.58 C: 48.93 ± 9.97	AWYC + WM	WM: quadruple therapy	4 wk	①②⑥
Chen D (2022)^[31]^	40/40	E: 24/16 C: 26/14	E: 37.07 ± 2.13 C: 36.72 ± 2.43	AWYC + WM	WM: triple therapy	4 wk	③④⑥
Xiong YF (2021)^[32]^	40/40	E: 25/15 C: 23/17	E: 44.49 ± 5.18 C: 44.77 ± 5.13	JHWKC + WM	WM: triple therapy	4 wk	①②③⑥
Xu LP (2019)^[33]^	46/46	E: 25/21 C: 26/20	E: 38.14 ± 6.03 C: 37.64 ± 5.81	JHWKC + WM	WM: quadruple therapy	1 mo	①②⑥
Zhao D (2015)^[34]^	49/49	E: 37/12 C: 36/13	E: 46.2 ± 3.3 C: 45.6 ± 2.3	JHWKC + WM	WM: triple therapy	14 d	①②⑤⑥
Mi CY (2022)^[35]^	46/46	E: 19/27 C: 21/25	E: 43.22 ± 5.07 C: 44.08 ± 5.11	JHWKC + WM	WM: quadruple therapy	14 d	①②⑥
Dai W (2018)^[36]^	52/52	E: 31/21 C: 29/23	E: 42.06 ± 5.38 C: 41.76 ± 5.13	WFCT + WM	WM: triple therapy	1 mo	①②③④
Zhou YY (2018)^[37]^	55/55	E: 30/25 C: 31/24	E: 39.25 ± 3.73 C: 39.32 ± 3.69	WFCT + WM	WM: triple therapy	10 d	①②③④⑥
Zheng CC (2020)^[38]^	30/30	33/27	46.1 ± 3.43	WFCT + WM	WM: triple therapy	14 d	①②⑤
Zhang X (2016)^[39]^	120/120	E: 50/70 C: 52/68	E: 43.1 ± 9.3 C: 41.3 ± 10.8	WFCT + WM	WM: triple therapy	4 wk	①②③④
Li LT (2015)^[40]^	40/40	E: 29/11 C: 28/12	E: 37.45 ± 3.45 C: 37.14 ± 3.09	WWSC + WM	WM: triple therapy	E: 63 d C: 56 d	①⑤⑥
Li SS (2019)^[41]^	43/43	E: 25/18 C: 21/22	E: 40.11 ± 6.52 C: 41.38 ± 5.71	WWSC + WM	WM: triple therapy	E: 1 mo C: 3 wk	①②⑥
Xu JY (2016)^[42]^	71/71	E: 41/30 C: 39/32	E: 43.1 ± 3.5 C: 42.3 ± 3.7	WWSC + WM	WM: triple therapy	14 d	①②⑤
Zhao GL (2020)^[43]^	43/42	E: 25/18 C: 23/19	E: 37.02 ± 5.11 C: 36.95 ± 5.07	WDAC + WM	WM: quadruple therapy	14 d	①
Liu M (2019)^[44]^	48/48	E: 36/12 C: 33/15	E: 50.3 ± 8.9 C: 52.0 ± 10.1	WDAC + WM	WM: proton pump inhibitor	6 wk	①②⑤⑥
Zheng XJ (2021)^[45]^	150/150	E: 77/73 C: 79/71	E: 47.2 ± 1.8 C: 46.5 ± 2.1	WDAC + WM	WM: quadruple therapy	4 wk	①②⑥
Wang J (2020)^[46]^	75/75	E: 44/31 C: 46/29	E: 55.41 ± 10.03 C: 54.98 ± 10.04	SGJYC + WM	WM: quadruple therapy	15 d	①②③④

AWYC = Anweiyang Capsule, BLWTG = Biling Weitong Granule, C = control group, E = experiment group, F = female, JHWKC = Jinghua Weikang Capsule, KFXS = Kangfuxin Solution, M = male, PWC = Pingwei Capsule, QWWTC = Qiwei Weitong Capsule, SGJYC = Shugan Jieyu Capsule, WDAC = Weidean Capsule, WFCT = Weifuchun Tablet, WM = Western medicine, WSG = Weisu Granule, WWSC = Wenweishu Capsule.

Outcomes: ① Clinical efficacy rate, ② *H pylori* eradication rate, ③ GAS levels, ④ motilin levels, ⑤ gastric ulcer recurrence rate, ⑥ adverse event rate.

### 3.3. Bias risk assessment

**Figure 2.**
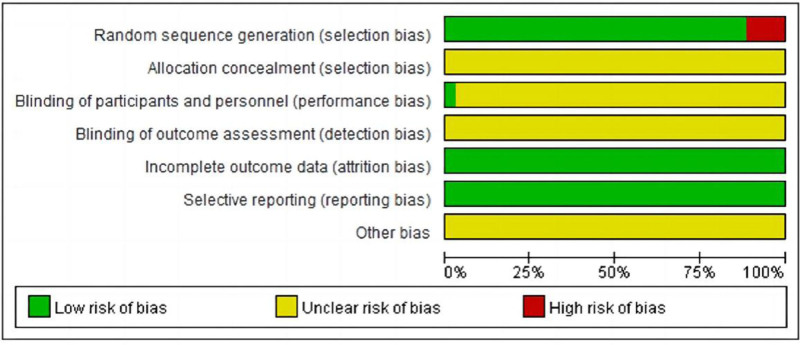
A graph showing the risk of bias.

**Figure 3.**
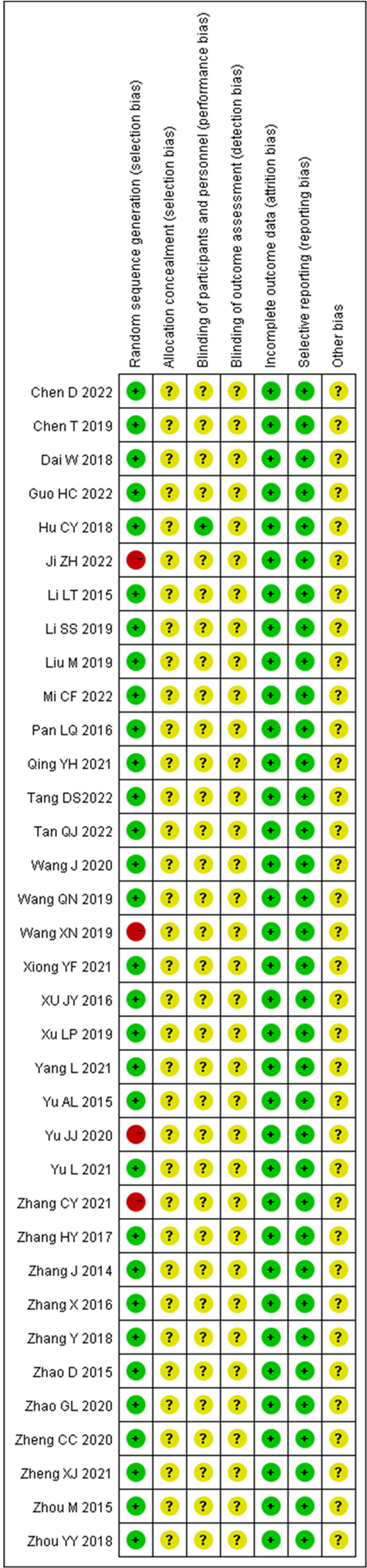
A summary of risk of bias. Green represents low risk, yellow represents unclear risk, and red represents high risk.

Figures 2 and 3 present the risk of bias assessment for entirety of the included trials, all of which were RCTs. Among the 31 studies, appropriate randomization generation techniques were employed, including computer-generated random numbers or tables of random numbers. Conversely, 4 studies used systematic, non-randomized methods during the sequence generation process, resulting in a substantially high risk of bias. Only 1 study described blinding for either the researchers or participants. Regarding incomplete outcome data and selective reporting, the studies were considered to have a “low risk of bias.” However, a majority of the test reports lacked sufficient information to precisely assess the results of “Allocation concealment” and “Blinding of outcome assessment,” eventually resulting in a significant proportion of unclear responses.

### 3.4. Network consistency and heterogeneity

We performed NMA on all 6 interconnected networks of evidence that examined clinical efficacy rate, *H pylori* eradication rate, GAS levels, MTL levels, GU recurrence rate, and adverse event rate outcomes individually. The absence of closed loops in the network graph precluded the need for inconsistency evaluation.^[44]^ Most of the studies included in our research had low heterogeneity (*I^2^* = 0%), necessitating the use of a random-effects model for the aggregated analyses.

### 3.5. Efficacy outcome

#### 3.5.1. Clinical efficacy rate

**Figure 4.**
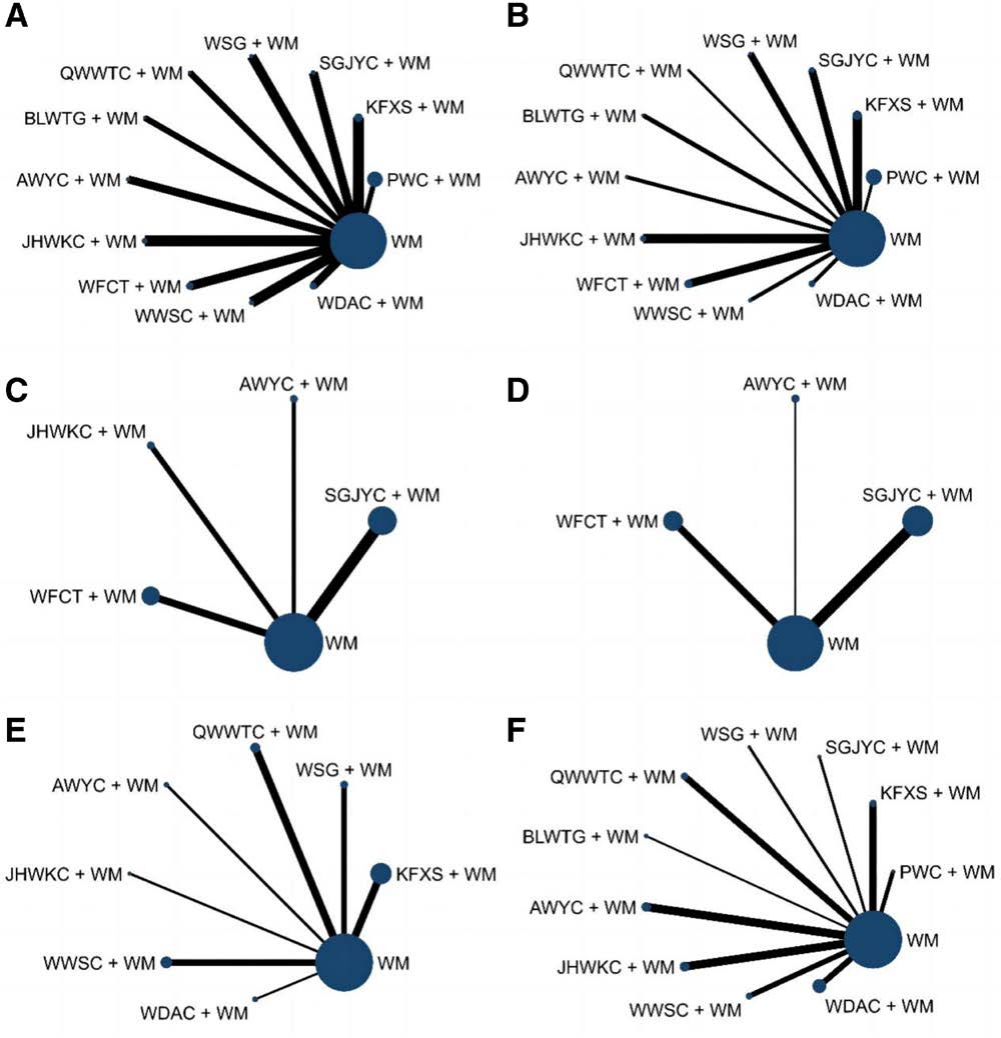
Network diagrams showing the outcome indicators, with the thickness of lines and size of circles proportional to the number of studies and participants, respectively. (A) Clinical efficacy rate; (B) *H pylori* eradication rate; (C) GAS levels; (D) MTL levels; (E) GU recurrence rate; (F) Adverse event rate. AWYC = Anweiyang Capsule, BLWTG = Biling Weitong Granule, GAS = gastrin, GU = gastric ulcer, JHWKC = Jinghua Weikang Capsule, KFXS = Kangfuxin Solution, MTL = motilin, PWC = Pingwei Capsule, QWWTC = Qiwei Weitong Capsule, SGJYC = Shugan Jieyu Capsule, WDAC = Weidean Capsule, WFCT = Weifuchun Tablet, WM = Western medicine, WSG = Weisu Granule, WWSC = Wenweishu Capsule.

A total of 34 RCTs, involving 4517 patients, provided extractable dichotomous data in terms of clinical efficacy rate.^[6,13–28,30–43,45–47]^ Upon pooling the data, we observed no heterogeneity (*I*^2^ = 0%). Figure 4A shows the network plot. The circle is proportional to the sample size, and lines between circles are direct comparative evidence, with thicker lines indicating more studies comparing CPM plus WM to WM.

**Figure 5.**
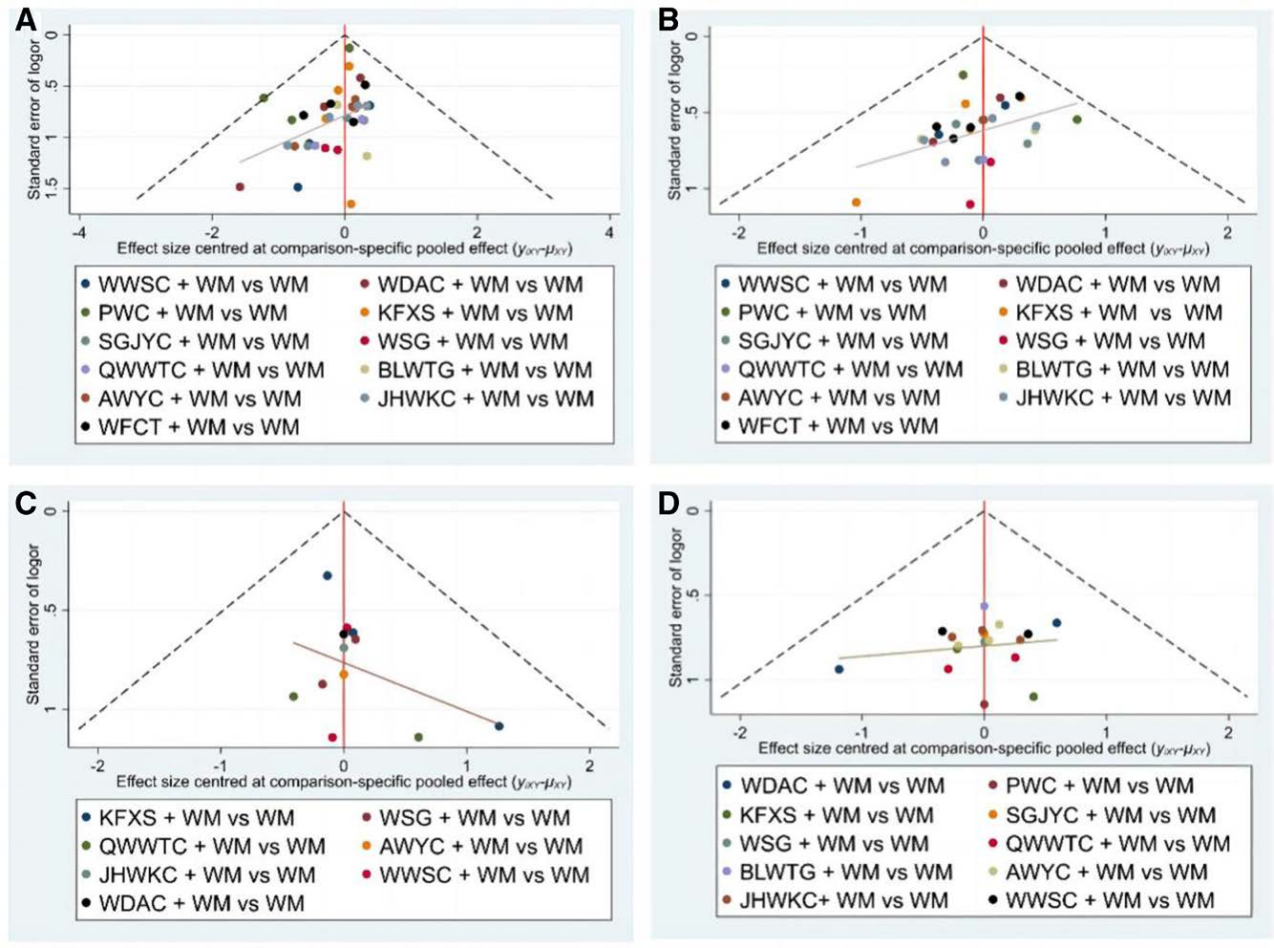
Funnel plots of outcome indicators. (A) Clinical efficacy rate; (B) *H pylori* eradication rate; (C) GU recurrence rate; (D) Adverse event rate. AWYC = Anweiyang Capsule, BLWTG = Biling Weitong Granule, GU = gastric ulcer; JHWKC = Jinghua Weikang Capsule, KFXS = Kangfuxin Solution, PWC = Pingwei Capsule, QWWTC = Qiwei Weitong Capsule, SGJYC = Shugan Jieyu Capsule, WDAC = Weidean Capsule, WFCT = Weifuchun Tablet, WM = Western medicine, WSG = Weisu Granule, WWSC = Wenweishu Capsule.

Publication bias is shown in Figure 5A. Funnel plot analysis was conducted to check for potential publication bias. The primary outcome funnel plot showed an asymmetrical distribution of data points around the midline, and the adjusted auxiliary line demonstrated non-perpendicular orientation to the midline, indicating potential publication bias among the recruited studies. Each study was excluded from the pooled-effect estimate in sensitivity analyses, the corresponding results of the current study were relatively robust.

**Figure 6.**
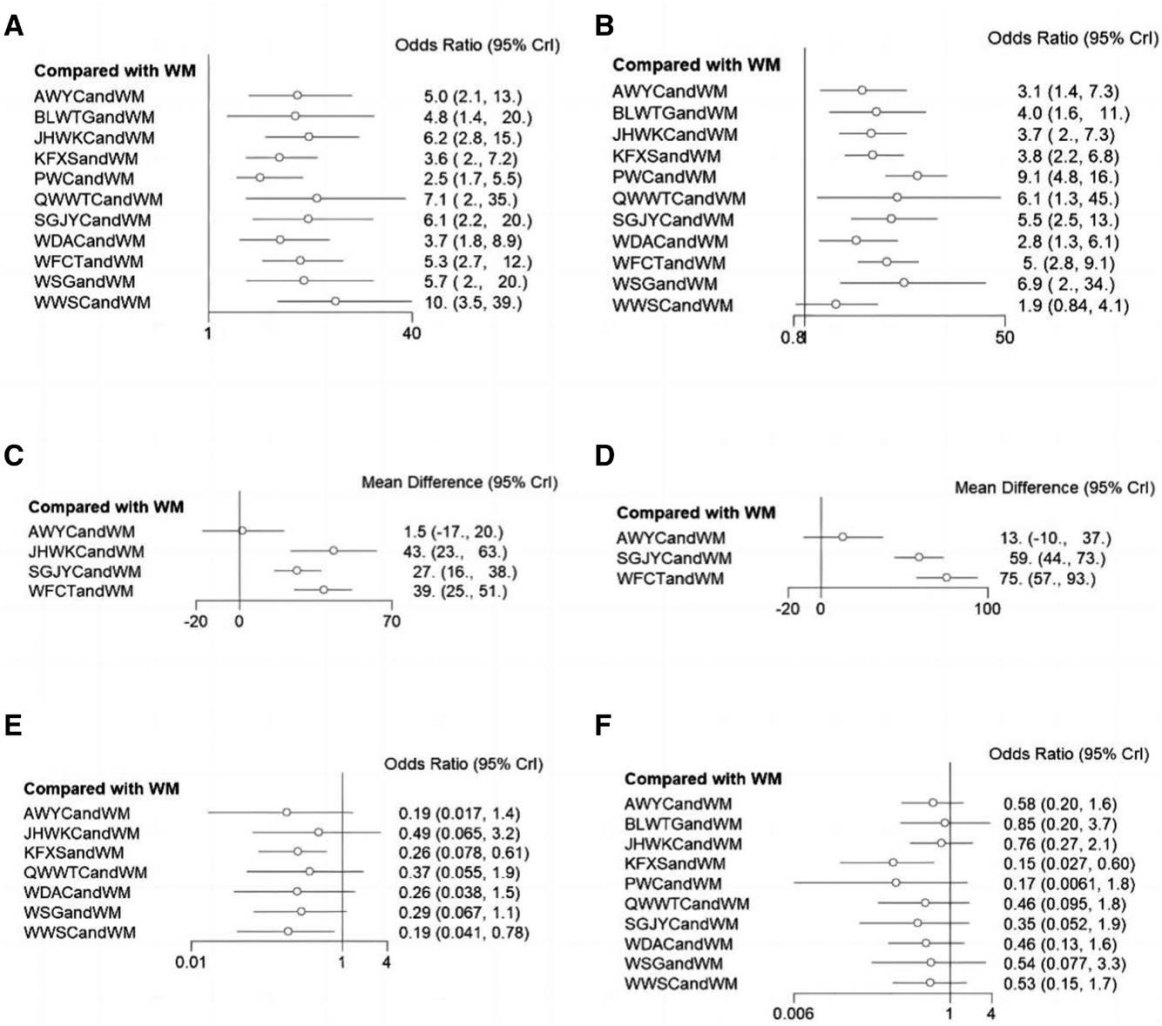
Forest plots of outcomes. (A) Clinical efficacy rate; (B) *H pylori* eradication rate; (C) GAS levels; (D) MTL levels; (E) GU recurrence rate; (F) Adverse event rate. AWYC = Anweiyang Capsule, BLWTG = Biling Weitong Granule, GAS = gastrin, GU = gastric ulcer, JHWKC = Jinghua Weikang Capsule, KFXS = Kangfuxin Solution, MTL = motilin, PWC = Pingwei Capsule, QWWTC = Qiwei Weitong Capsule, SGJYC = Shugan Jieyu Capsule, WDAC = Weidean Capsule, WFCT = Weifuchun Tablet, WM = Western medicine, WSG = Weisu Granule, WWSC = Wenweishu Capsule.

Statistically significant differences were noted between the combined treatment of CPMs and WM as well as WM alone (Fig. 6A). The results of the NMA indicated that the combination of proprietary CPMs and WM had superior performance compared to WM alone. The combined application of PWC and WM demonstrated lower clinical efficacy, unlike the combined use of JHWKC and WM, as well as WWSC and WM. The ORs and corresponding 95% CIs were 0.40 [0.18, 0.88], 0.45 [0.23, 0.88], and 0.29 [0.10, 0.85], respectively (Table 2).

**Table 2:**
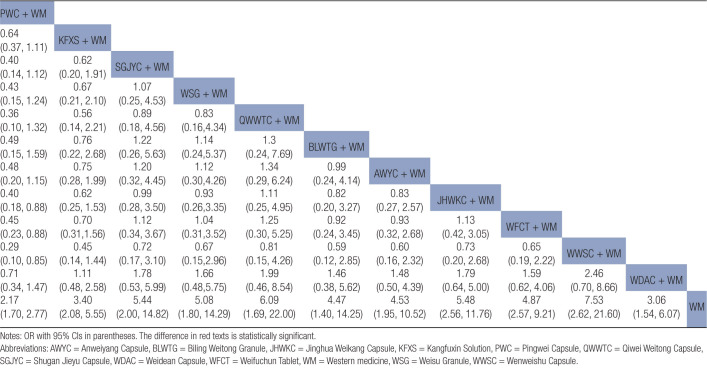
Results (OR, 95% CI) of network meta-analysis for clinical efficacy rate.

**Figure 7.**
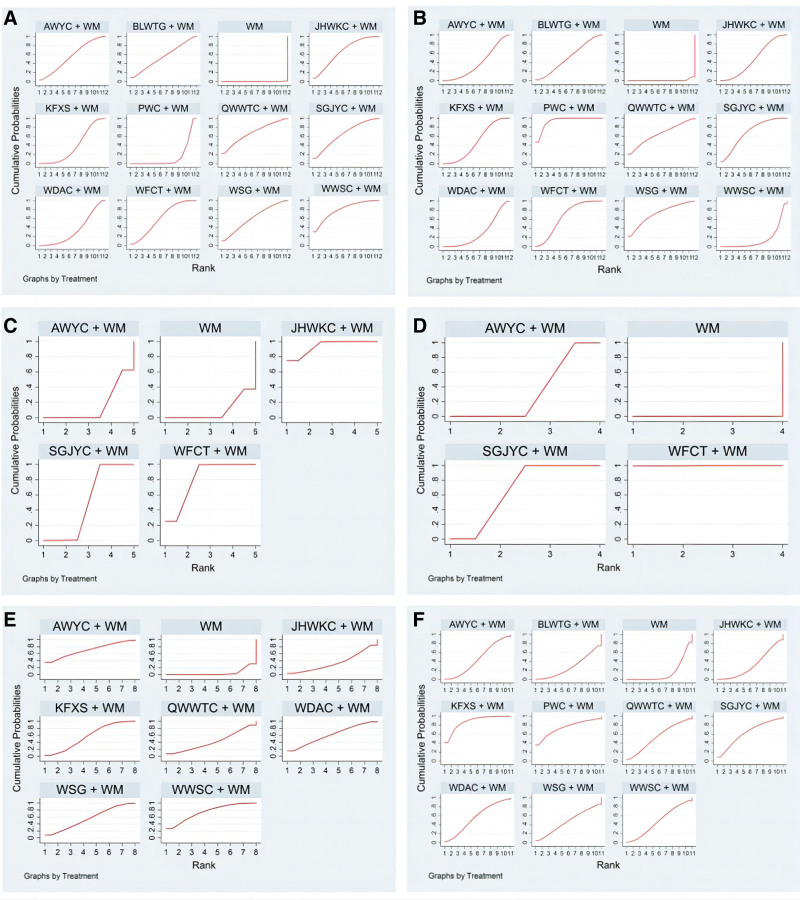
Curve diagrams of SUCRA showing outcome indicators. (A) Clinical efficacy rate; (B) *H pylori* eradication rate; (C) GAS levels; (D) MTL levels; (E) GU recurrence rate; (F) Adverse event rate. AWYC = Anweiyang Capsule, BLWTG = Biling Weitong Granule, GAS = gastrin, GU = gastric ulcer, JHWKC = Jinghua Weikang Capsule, KFXS = Kangfuxin Solution, MTL = motilin, PWC = Pingwei Capsule, QWWTC = Qiwei Weitong Capsule, SGJYC = Shugan Jieyu Capsule, WDAC = Weidean Capsule, WFCT = Weifuchun Tablet, WM = Western medicine, WSG = Weisu Granule, WWSC = Wenweishu Capsule.

The probability of a high to low clinical effective rate for CPMs combined with WM was ranked based on the SUCRA as follows: WWSC + WM (79.3%) > QWWTC + WM (68.4%) > SGJYC + WM (65.3%) > JHWKC + WM (66.9%) > WSG + WM (61.3%) > WFCT + WM (60.9%) > AWYC + WM (55.7%) > BLWTG + WM (54.9%) > KFXS + WM (38.5%) > WDAC + WM (34.2%) > PWC + CT (14.5%) > WM (0.1%) (Fig. 7A).

#### 3.5.2. H pylori eradication rate

A total of 28 RCTs, involving 4090 patients, provided extractable dichotomous data in terms of *H pylori* eradication rate.^[6,13–23,25,26,28–30,33–39,41–43,45]^ When data were pooled, there was no heterogeneity (*I*^2^ = 0%). The network plot is shown in Figure 4B.

Publication bias is shown in Figure 5B. The majority of effect sizes were situated in the central region of the funnel plot and exhibited a relatively uniform distribution on either side of mean effect size. The adjusted auxiliary line displayed a non-perpendicular orientation to the midline, suggesting the presence of publication bias. Each study was excluded from the pooled-effect estimate in sensitivity analyses, and the corresponding results of the current study were relatively robust.

The forest figure revealed that except for WWSC, a statistically significant difference was found when combining other CPMs with WM and WM alone (Fig. 6B). The findings of the NMA revealed that combining PWC and WM exhibited superior efficacy in the eradication of *H pylori* unlike the combinations of KFXS and WM, AWYC and WM, JHWKC and CT, WWSC and WM, and WDAC and WM. The ORs and corresponding 95% CIs were 2.55 [1.29, 5.04], 3.02 [1.25, 7.03], 2.59 [1.2, 5.59], 5.03 [2.14, 11.83], and 3.45 [1.52, 7.81], respectively. The efficacy of SGJYC combined with WM and WFCT combined with WM in the eradication of *H pylori* surpassed that of WWSC combined with WM, as shown by the ORs and 95% CIs of 2.88 [1, 8.28] and 2.63 [1.08, 6.42], respectively (Table 3).

**Table 3:**
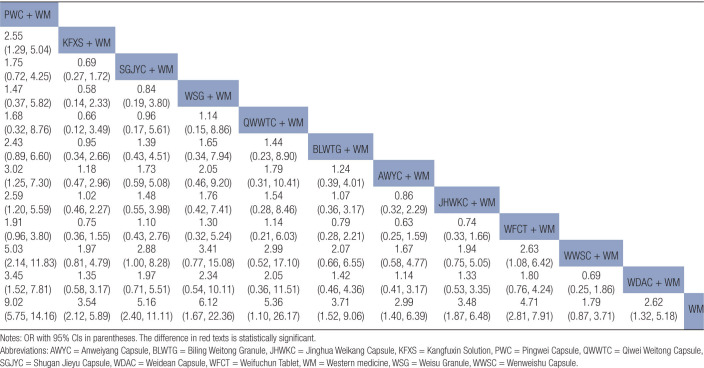
Results (OR, 95% CI) of network meta-analysis for *H pylori* eradication rate.

The probability of a high to low *H pylori* eradication rate in CPMs combined with WM was ranked based on the SUCRA as follows: PWC + WM (93.1%) > WSG + WM (72.4%) > SGJYC + WM (69%) > WFCT + WM (65.7%) > QWWTC + WM (65.4%) > BLWTG + WM (51.1%) > KFXS + WM (48%) > JHWKC + WM (47.1%) > AWYC + WM (38.8%) > WDAC + WM (32.3%) > WWSC + WM (16.2%) > WM (0.8%) (Fig. 7B).

#### 3.5.3. GAS levels

Further, 9 RCTs, incorporating 715 patients, provided extractable continuous data in terms of GAS levels.^[14,16,29–31,35,36,38,47]^ When data were pooled, there was moderate heterogeneity (*I*^2^ = 43%). The network plot is shown in Figure 4C. A forest map showed that the combined treatment of other CPMs and WM differed statistically significantly from WM treatment alone except for AWYC (Fig. 6C). The probability of decreasing GAS levels to CPM combined with WM was ranked according to the SUCRA from high to low as follows: JHWKC + WM (93.6%) > WFCT + WM (81.2%) > SGJYC + WM (50.1%) > AWYC + WM (15.6%) > WM (9.4%) (Fig. 7C).

#### 3.5.4. MTL levels

A total of RCTs, with 635 patients, provided extractable continuous data in terms of MTL levels.^[14,29–31,35,36,38,47]^ When the data were pooled, there was no heterogeneity (*I*^2^ = 0%). The network plot is presented in Figure 4D. The forest map showed that, except for AWYC, there were statistically significant differences between the combined treatment of other CPMs and WM as well as WM alone (Fig. 6D). The probability of decreasing MTL levels to CPMs combined with WM was ranked according to the SUCRA from high to low as follows: WFCT + WM (99.9%) > SGJYC + WM (66.8%) > AWYC + WM (33.3%) > WM (0%) (Fig. 7D).

### 3.6. Safety outcome

#### 3.6.1. GU recurrence rate

A total of 13 RCTs, involving 1221 patients, provided extractable dichotomous data in terms of GU recurrence rate.^[6,19–21,26,33,37,39–41,43,46,47]^ There was no heterogeneity when the data were pooled (*I*^2^ = 0%). Figure 4E shows the network plot, and Figure 5C shows the publication bias. The primary outcome funnel plot displayed an asymmetrical distribution of data points around the midline; the adjusted auxiliary line demonstrated non-perpendicular orientation to the midline, indicating possible publication bias among the studies analyzed. Each study was excluded from the pooled-effect estimate in sensitivity analyses. The corresponding results of the current study were relatively robust.

Forest plot results revealed that KFXS and WM combination as well as the WWSC and WM combination had statistically significant variations in producing GU recurrence unlike WM treatment alone (Fig. 6E). The results of NMA indicated that KFXS, WSG, WWSC, and WDAC, when combined with WM, yielded reduced rates of GU recurrence unlike WM alone, as shown by their respective OR and 95% CI values of 0.30 [0.18, 0.52], 0.31 [0.11, 0.85], 0.21 [0.08, 0.59], and 0.27 [0.08, 0.92] (Table 4).

**Table 4:**
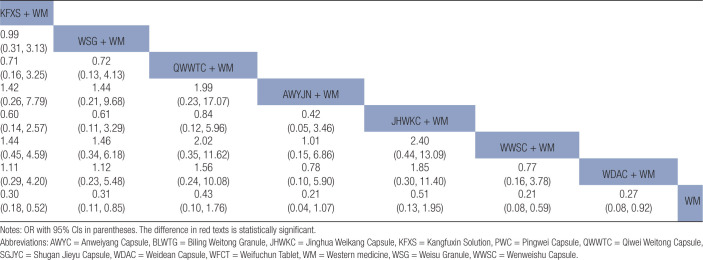
Results (OR, 95% CI) of network meta-analysis for GU recurrence rate.

The probability of GU recurrence rate in CPMs combined with WM was ranked according to the SUCRA by likelihood from low to high as follows: WWSC + WM (74.5%) > AWYC + WM (70.2%) > WDAC + WM (61%) > KFXS + WM (57.1%) > WSG + WM (56.1%) > QWWTC + WM (41.7%) > JHWKC + WM (34.4%) > WM (5%) (Fig. 7E).

#### 3.6.2. Adverse event rate

A total of 20 RCTs, involving 1772 patients, provided extractable dichotomous data in terms of adverse event rate outcomes.^[6,13,14,16,17,19,20,24,26,27,30,31,33,34,36,39,42,45–47]^ When the data were pooled, there was no heterogeneity (*I*^2^ = 0%). The network plot is shown in Figure 4F.

Publication bias is shown in Figure 5D. Most effect sizes were located at the central part of the funnel plot and were uniformly distributed on either side of the mean effect size. Additionally, the adjusted auxiliary line showed a perpendicular orientation to the midline, indicating no publication bias. The forest plots and NMA results confirmed that the KFXS and WM combination had a statistically significant effect compared to WM alone, as shown by the OR and 95% CI values of 0.18 [0.05, 0.64] (Figure 6F and Table 5).

**Table 5:**
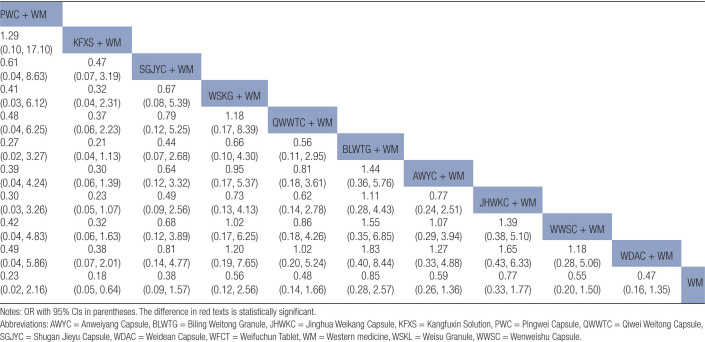
Results (OR, 95% CI) of network meta-analysis for adverse event rate.

The probability of adverse event rate for CPMs combined with WM was determined based on the SUCRA by likelihood from low to high as follows: KFXS + WM (87.6%) > PWC + WM (74.3%) > SGJYC + WM (62.6%) > WDAC + WM (55.9%) > QWWTC + WM (53.8%) > WWSC + WM (48.2%) > WSG + WM (46.8%) > AWYC + WM (45%) > JHWKC + WM (32.3%) > BLWTG + WM (28.3%) > WM (15.2%) (Fig. 7F).

#### 3.6.3. Adverse event analysis

Table 6 comprehensively lists the adverse events based on our comprehensive literature review. The most common adverse events included infection, gastrointestinal symptoms (diarrhea, nausea, and vomiting), and dermatosis (rash). Unlike CPMs combined with the WM group, the WM alone group had a higher incidence of adverse events.

**Table 6:** Summary of adverse events.

Adverse reaction	RCT															
	Pan LQ (2016)^[14]^	Tan DS (2022)^[18]^	Tan QJ (2022)^[23]^	Chen T (2019)^[27]^	Hu CY (2018)^[6]^	Ji ZH (2022)^[28]^	Zhang CY (2021)^[30]^	Guo HC (2022)^[17]^	Zheng XJ (2021)^[45]^	Xu LP (2019)^[33]^	Zhou YY (2018)^[37]^	Li SS (2019)^[41]^	Li LT (2015)^[40]^	Xiong YF (2021)^[32]^	Mi CY (2022)^[35]^	Liu M (2019)^[44]^
Nausea	E: 2/37 C: 4/37	**/**	E: 3/34 C: 5/34	**/**	E: 3/43 C: 3/43	E: 3/48 C: 1/48	E: 1/42 C: 2/42	C: 2/36	E: 1/150 C: 4/150	E: 2/46 C: 2/46	E: 3/55 C: 2/55	E: 3/43 C: 5/43	E: 2/40 C: 2/40	**/**	C: 1/46	**/**
Rash	**/**	**/**	**/**	E: 1/42 C: 1/42	**/**	E: 1/48 C: 2/48	C: 1/42	**/**	**/**	**/**	**/**	**/**	**/**	E: 1/40 C: 1/40	E: 1/46	**/**
Constipation	**/**	E: 1/36 C: 2/36	**/**	E: 1/42 C: 2/42	**/**	**/**	E: 1/42 C: 1/42	**/**	**/**	**/**	**/**	**/**	**/**	**/**	**/**	**/**
Diarrhea	**/**	E: 1/36 C: 3/36	**/**	C: 1/42	C: 4/43	E: 2/48 C: 3/48	E: 1/42 C: 1/42	C: 2/36	E: 1/150 C: 3/150	E: 1/46 C: 1/46	**/**	C: 1/40	C: 1/40	**/**	**/**	**/**
Poor appetite	**/**	**/**	**/**	**/**	**/**	**/**	**/**	E: 1/36 C: 3/36	**/**	**/**	**/**	**/**	**/**	**/**	**/**	**/**
Headache	**/**	**/**	**/**	**/**	**/**	**/**	**/**	**/**	E: 1/150 C: 2/150	E: 1/46 C: 1/46	**/**	**/**	**/**	E: 1/40 C: 1/40	E: 2/46	**/**
lethargy	**/**	C: 1/36	**/**	**/**	**/**	**/**	**/**	**/**	**/**	**/**	**/**	E: 2/40 C: 2/40	E: 2/40 C: 2/40	**/**	**/**	E: 1/48
Dizziness	**/**	**/**	**/**	**/**	**/**	**/**	**/**	**/**	**/**	**/**	**/**	**/**	**/**	E: 1/40	**/**	E: 1/48
Fatigue	**/**	**/**	**/**	**/**	**/**	**/**	**/**	**/**	**/**	**/**	E: 2/55 C: 1/55	**/**	**/**	E: 1/40 C: 2/40	**/**	**/**
Allotriogeustia	**/**	**/**	**/**	**/**	**/**	E: 1/48 C: 2/48	**/**	**/**	**/**	C: 1/46	E: 2/55 C: 1/55	**/**	**/**	**/**	**/**	E: 1/48
Gastrointestinal reaction	**/**	**/**	**/**	**/**	**/**	**/**	**/**	**/**	**/**	**/**	**/**	**/**	**/**	**/**	E: 3/46 C: 4/46	**/**
Insomnia	**/**	**/**	**/**	C: 1/42	**/**	**/**	**/**	**/**	C: 2/150	**/**	**/**	**/**	**/**	**/**	**/**	**/**
Tinnitus	**/**	**/**	**/**	**/**	**/**	**/**	**/**	**/**	**/**	**/**	**/**	**/**	**/**	**/**	**/**	C: 1/48
Facial puffiness	**/**	**/**	C: 1/34	**/**	**/**	**/**	**/**	**/**	**/**	**/**	**/**	**/**	**/**	**/**	**/**	**/**
Tachycardia	**/**	**/**	C: 1/34	**/**	**/**	**/**	**/**	**/**	**/**	**/**	**/**	**/**	**/**	**/**	**/**	**/**

C = control group, E = experiment group, RCT = randomized controlled trial.

### 3.7. Cluster analysis

**Figure 8.**
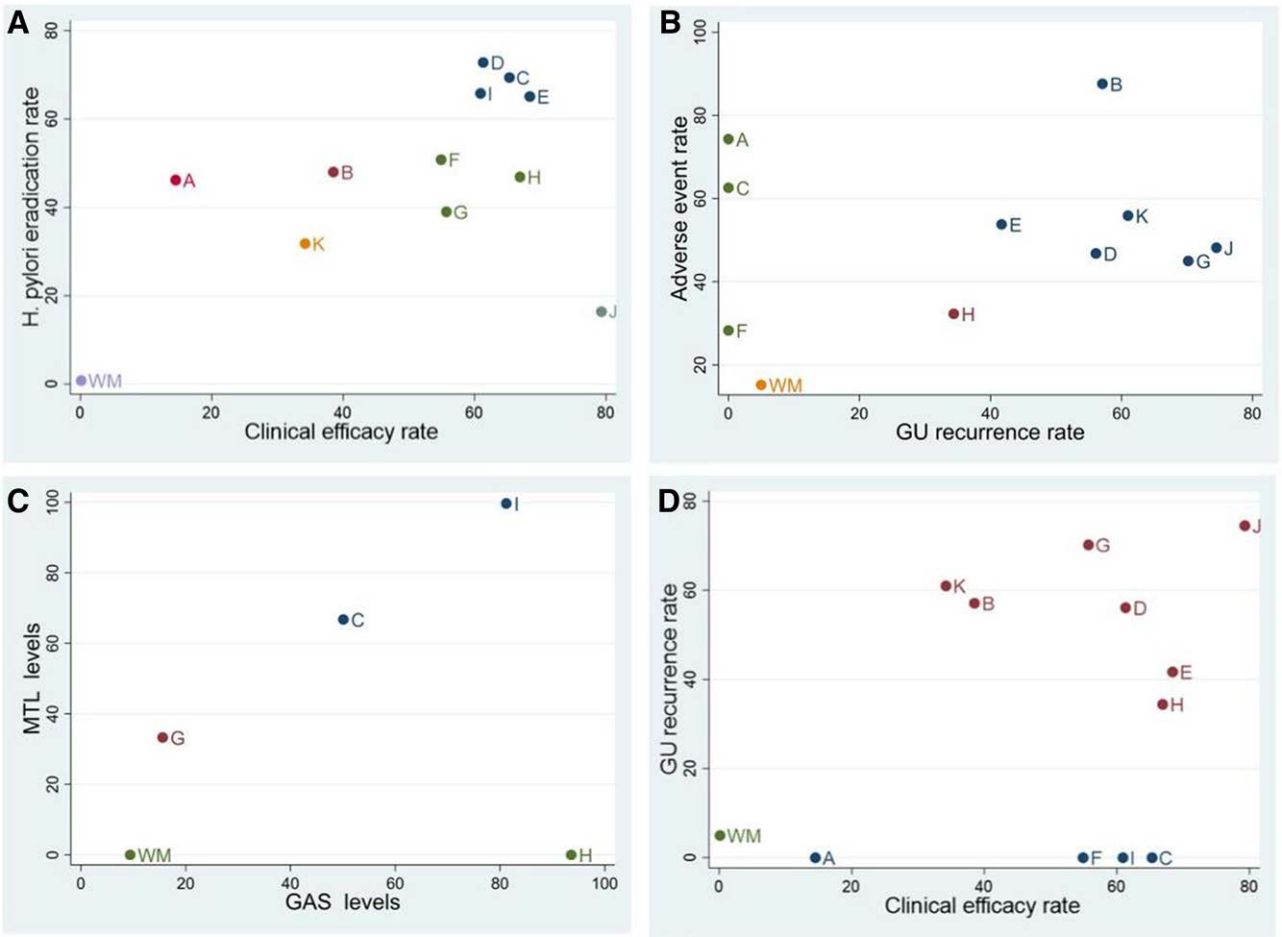
Plots for cluster analysis. (A) Clinical efficacy rate and *H pylori* eradication rate; (B) GU recurrence rate and adverse event rate; (C) GAS levels and MTL levels. (D) Clinical effectiveness rate and GU recurrence rate. Interventions located in the upper right corner indicate optimal therapies for 2 different outcomes, as follows: A, PWC + WM; B, KFXS + WM; C, SGJYC + WM; D, WSG + WM; E, QWWTC + WM; F, BLWTG + WM; G, AWYC + WM; H, JHWKC + WM; I, WFCT + WM; J, WWSC + WM; K, WDAC + WM; WM, Western medicine.

Cluster analyses of clinical efficacy and secondary outcomes were performed to assess the overall efficacy of 12 treatments. The findings of the 2-dimensional clustering analysis suggested that WWSC and WM combination, located at the farthest position from the zero point, yielded the most significant improvement in clinical efficacy. The WM regimen in isolation ranked lowest on eradication rate of *H pylori*, whereas PWC combined with WM was the most effective (Fig. 8A). Moreover, the combination of KFXS and WM was the optimal therapeutic approach in mitigating both the *H pylori* eradication rate and adverse event rate (Fig. 8B). WFCT combined with WM was the preferred treatment for decreasing GAS and MTL levels (Fig. 8C). WWSC and WM combination displayed superior clinical efficacy and emerged as the preferred treatment for reducing the recurrence rate of GU (Fig. 8D).

## 4. Discussion

*H pylori*-related GU is among the most common gastrointestinal disorders globally, with upper abdominal discomfort, often accompanied by symptoms including heartburn, acid reflux, hiccups, or decreased appetite.^[48]^ Generally, conventional WMs are the most commonly used treatment for *H pylori*-related GU. With a more thorough mechanistic understanding of GU, *H pylori* infection has been identified as the most critical pathogenic factor.^[49]^ *H pylori* is a highly mobile bacterium that can penetrate the gastric wall using its flagella and secrete proteins to neutralize gastric acid. This increases the pH level of gastric juice, consequently creating an environment conducive to *H pylori* colonization and reproduction.^[50]^ Under typical physiological circumstances, the majority of pepsinogen secreted by gastric mucosa enters the stomach and undergoes conversion into pepsin through gastric acid activation. *H pylori* infection can stimulate gastric mucosal epithelial cells, activate neutrophils, and promote proteolytic enzyme production. This can result in the occurrence of gastric mucosal reverse digestion, ulcers, and gastric cancer in severe cases. The activation of the nuclear factor κB (NF-κB) signal transduction pathway by *H pylori* is involved in the occurrence, development, and repair of GU. This process disrupts the ion exchange system of gastric mucosa, altering the pH environment in the stomach. Consequently, the primary therapeutic targets for promoting the treatment of GU include inhibiting excessive gastric acid secretion, reducing pepsin activity, eradicating *H pylori*.^[40]^

The current predominant approach in WMs for addressing *H pylori*-associated GU involves the administration of triple or quadruple therapy for eradication. However, this approach has significant shortcomings, including high costs, significant drug resistance, increased toxicity, and side effects. In contrast, TCM emphasizes comprehensive regulation and enhancement of the body’s innate immunity while at the same time exerting direct inhibitory effects on *H pylori*.^[51,52]^ In recent years, CPMs have been broadly used in treating *H pylori*-related GU due to their cost-effectiveness, minimal adverse effects, and consistent therapeutic benefits. However, as to which CPM has the best therapeutic effect against *H pylori*-related GU remains inconclusive.^[53]^

For the first time, this NMA investigates the optimal efficacy and safety for 11 CPMs combined with WMs in GU with *H pylori* infection. This work sought to provide a reference for accurate and rigorous clinical use of CPMs and the design of subsequent related clinical studies.^[54]^ The efficacy outcomes revealed that 11 CPMs could excellently improve clinical efficacy and the rate of *H pylori* eradication over WM, with a statistically significant difference. WWSC combined with WM displayed the most favorable clinical efficiency, as shown by its high SUCRA value of 79.3%. PWC and WM combination yielded the most significant eradication rate of *H pylori*, with a corresponding SUCRA value of 93.1%. The combination of WM with SGJYC, AWYC, JHWKC, and WFCT effectively decreased the GAS levels. JHWKC displayed the highest efficacy with a SUCRA value of 93.6%. The use of WM combined with SGJYC, AWYC, and WFCT effectively lowered MTL levels. Furthermore, WFCT combined with WM displayed the most optimal performance in reducing GAS and MTL levels, with a SUCRA value reaching up to 99.9%. Safety outcomes showed a significantly lower incidence of *H pylori* eradication with CPMs combined with WM than with WM alone. WWSC and WM combination displayed a superior efficacy in reducing the recurrence rate of GU, as shown by its significant SUCRA value of 74.5%. The amalgamation of KFXS and WM demonstrated superior efficacy in mitigating the incidence of adverse events, as substantiated by its great SUCRA value of 87.6%.

According to the pharmacopeia of the People’s Republic of China, WWSC comprises *Codonopsis* Radix, *AconitI Lateralls* Radix Praeparata, *Astragalus* Radix, *Cinnamoi* Cortex, *Dioscoreae* Rhizoma, *Citri Reticulatae* Pericarpium, *Amomi* Fructus, *Crataegi* Fructus, *Mume* Fructus, *Atractylodis Macrocephalae* Rhizoma, *Psoraleae* Fructus, and *Cistanches* Herba.^[43]^ Modern pharmacological research has shown that *AconitI Lateralls* Radix Praeparata is popular for its antiarrhythmic properties, myocardium protection, hypoxia tolerance improvement, anti-inflammatory effects, and analgesic effects, whereas *Codonopsis* radix is known to improve the conditions of spleen and stomach weakness, promote fluid, and invigorate Qi. Astragalus radix can improve immune function, protect the liver, exhibit diuretic properties, as well as exert antiaging, anti-stress, antihypertensive, and antibacterial effects. Both *Astragalus* radix and *Codonopsis* radix ginseng can enhance hematopoiesis and protein synthesis, thereby promoting the regeneration of mucosal cells and gland cells.^[6]^ *Atractylodis Macrocephalae* Rhizoma can warm and replenish the body, improve the body’s resistance, and encourage the Qi machine. *Cinnamoi Cortex* is known for its calming and cooling properties, as well as its capacity to lower blood pressure, prevent schistosomiasis, and strengthen the stomach, with bactericidal effects. *Dioscoreae* Rhizoma has beneficial properties including tonifying the spleen and stomach, promoting fluid balance, supporting lung function, as well as tonifying the kidney and astringing essence. On the other hand, *Citri Reticulatae Pericarpium* can regulate Qi, invigorate the spleen, dispel dampness, and eliminate phlegm. Moreover, the *Amomi Fructus* is acknowledged for its dampness-dispelling, appetite-stimulating, spleen-warming, diarrhea-stopping, Qi-regulating, and fetus-calming properties. *Crataegi Fructus* promotes Qi circulation, alleviates stasis, and fortifies the digestive and spleen functions. Cistanches Herba augments the essence and blood as well as invigorates the kidney, Yang. Mume Fructus is a sour and astringent product that focuses on astringent intestine to prevent diarrhea. Psoraleae is a warm product that can tonify the kidney, solid essence shrinks urine, and help Yang stop diarrhea.^[55]^ Based on contemporary pharmacological research, WWSC exhibits anti-*H pylori* effects and can restore damaged gastric mucosa at the same time suppressing ulcer development. Furthermore, it can augment pepsin activity, elicit the secretion of digestive enzymes and fluids, improve digestion in patients, as well as stimulate gastric mucosal microcirculation and motility.^[56]^ Several animal experiments have shown that the WWSC can impede NF-κB signaling pathway activation in rats, hence reducing NF-κBp65, IκBα, COX_2_ protein levels, and TNF-α and IL-6 inflammatory factors.^[57]^

PWC comprises *Atractylodis* Rhizoma, *Fritillariae Thunbergii* Bulbus, *Aurantii* Fructus, *Magnoliae Officinalis* Cortex, *Bupleuri* Radix, *Citri Reticulatae* Pericarpium, *Fritillariae Thunbergii* Bulbus, *Taraxaci* Herbapi, *Coptidis* Rhizoma, Sepiae Endoconcha, Galli Gigerii Endothelium Corneum, *Corydalis* Rhizoma, *Aucklandia* Radix, *Bletillae* Rhizoma, and *Sparganii* Rhizoma.^[58]^ PWC is formulated based on the principles of pingwei powder, as described in the “prescription of peaceful benevolent dispensary” during the Song Dynasty. *Atractylodis* Rhizoma is popular for its pungent fragrance and bitter warmth, which can effectively alleviate dampness, strengthen the spleen, promote Qi, and regulate the stomach.^[23]^ Similarly, *Magnoliae Officinalis* Cortex has bitterness, warmth, fragrance, and Qi circulation, whereas Citri Reticulatae Pericarpium helps regulate Qi and promote blood flow. *Coptidis* Rhizoma and *Taraxaci* Herbapi are effective in clearing heat and fire as well as reducing dampness and heat accumulation in gastrointestinal tract. *Aurantii* Fructus, *Corydalis* Rhizoma, *Aucklandia* Radix, *Bupleuri* Radix, and *Sparganii* Rhizoma can eliminate Qi knots and soothe the liver and stomach.^[59]^ White *Bletillae* Rhizoma, Sepiae Endoconcha, and *Fritillariae Thunbergii* Bulbus inhibit acid secretion and relieve pain. *Fritillariae Thunbergii* Bulbus nourishes the blood and softens the liver; Galli Gigerii Endothelium Corneum helps in digestion and strengthens the stomach. Experimental findings show that the administration of PWC improves the defense mechanism of the gastric mucosa, blocks gastric acid secretion, mitigates inflammation, and alleviates pain in a model of GU.^[60]^

## 5. Limitations

This study has worth mentioning limitations: The included studies should have higher methodological quality; 4 of the 35 RCTs did not explain the method of generating random sequences, and only 1 study mentioned allocation concealment or blinding. Despite the absence of geographical limitations in study selection, all eligible studies were conducted and published in Chinese journals within mainland China, thereby limiting the generalizability of the findings. There was a lack of direct comparison of 2 or more CPMs in most of the included RCTs since majority were control experiments. The sample sizes included in the RCTs varied in size, and the small sample size may have contributed to the lack of significant difference. Therefore, increasing the sample size and balancing the number of RCTs focused on different kinds of CPMs would strengthen the statistical power and credibility of the NMA. Besides, additional RCTs with higher methodological quality are necessary to validate the value of CPMs combined with WMs in the treatment of patients with *H pylori*-related GU. Despite the limitations, this study, for the first time, attempts to assess the efficacy and safety of CPMs as well as the integration of Chinese and WMs in the management of *H pylori*-induced GU; we performed an NMA to rank the clinical efficacy rate, *H pylori* eradication rate, GAS and MTL levels, GU recurrence rate, and incidence of adverse events.

## 6. Conclusion

In summary, this NMA presents a comprehensive and integrated evaluation and summary of the findings of CPMs used for the treatment of *H pylori*-related GU. The application of CPMs in conjunction with WM has been found to yield superior results in enhancing clinical outcomes and mitigating GU recurrence rates compared with WM monotherapy. Administration of WWSC combined with WM was found to be the optimal therapeutic approach. In terms of the *H pylori* eradication rate, PWC combined with WM was superior to WM alone. The combination of WFCT and WM showed the highest efficacy in diminishing GAS and MTL levels. For safety outcomes, the combination of KFXS and WM was the most effective therapeutic strategy for suppressing the incidence of adverse events. Our NMA results have important implications in clinical research and practice. To validate these findings, future investigations should employ larger sample sizes and multicenter RCTs to conduct real-world clinical studies.

## Author contributions

**Conceptualization:** Meiqi Zhong.

**Data curation:** Meiqi Zhong, Qifang Sun, Baoping Ren.

**Formal analysis:** Meiqi Zhong, Chang Yu, Shunhua Zhou, Qing Gao.

**Methodology:** Meiqi Zhong, Xiaojuan Wang, Chengzhi Yuan, Jing Lu.

**Software:** Meiqi Zhong, Qinghua Peng, Meiyan Zeng, Houpan Song.

**Resources:** Qinghua Peng, Meiyan Zeng, Houpan Song.

**Writing – original draft:** Meiqi Zhong.

**Writing – review & editing:** Qinghua Peng, Meiyan Zeng, Houpan Song.

## References

[1] Li L Du Y Wang Y He N Wang B Zhang T. Atractylone alleviates ethanol-induced gastric ulcer in rat with altered gut microbiota and metabolites. J Inflamm Res. 2022;15:4709–23. 35996682 10.2147/JIR.S372389 PMC9392477

[2] Byeon S Oh J Lim JS Lee JS Kim J-S. Protective effects of Dioscorea Batatas flesh and peel extracts against ethanol-induced gastric ulcer in mice. Nutrients. 2018;10:1680. 30400615 10.3390/nu10111680 PMC6266015

[3] Shah SC Tepler A Chung CP. Host genetic determinants associated with Helicobacter pylori eradication treatment failure: a systematic review and meta-analysis. Gastroenterology. 2021;161:1443–59. 34358488 10.1053/j.gastro.2021.07.043 PMC8545829

[4] Hu XC Tian WR Liu JX. Progress of experimental research on the treatment of gastric ulcer by traditional Chinese medicine. J Ningxia Med Univ. 2014;36:825–8.

[5] Bhattamisra SK Yan VL Y Lee C K. Protective activity of geraniol against acetic acid and Helicobacter pylori-induced gastric ulcers in rats. J Tradit Complement Med. 2018;9:206–14. 31193983 10.1016/j.jtcme.2018.05.001 PMC6544613

[6] Hu CY Xiong Y. Effect of Anweiyang capsule combined with quadruple therapy on Helicobacter pylori positive gastric ulcer. Clin Res Pract. 2018;3:140–2.

[7] Xia RY Liang WX Liu CH. Economic evaluation of seven oral Chinese patent medicines combined with triple therapy for Helicobacter Pylori related peptic ulcer and gastritis. Chin J Pharm Econ. 2022;17:29–38.

[8] Gao Y Shi S Li M. Statistical analyses and quality of individual participant data network meta-analyses were suboptimal: a cross-sectional study. BMC Med. 2020;18:120. 32475340 10.1186/s12916-020-01591-0 PMC7262764

[9] Dias S Sutton AJ Welton NJ Ades AE. Evidence synthesis for decision making 3: heterogeneity--subgroups, meta-regression, bias, and bias-adjustment. Med Decis Making. 2013;33:618–40. 23804507 10.1177/0272989X13485157 PMC3704206

[10] Hamra G MacLehose R Richardson D. Markov chain Monte Carlo: an introduction for epidemiologists. Int J Epidemiol. 2013;42:627–34. 23569196 10.1093/ije/dyt043 PMC3619958

[11] Shim S Yoon BH Shin IS Bae J-M. Network meta-analysis: application and practice using Stata. Epidemiol Health. 2017;39:e2017047. 29092392 10.4178/epih.e2017047 PMC5733388

[12] Rucker G Schwarzer G. Ranking treatments in frequentist network meta-analysis works without resampling methods. BMC Med Res Methodol. 2015;15:58. 26227148 10.1186/s12874-015-0060-8 PMC4521472

[13] Zhang J Fu W Wang LD. Pingwei capsule combined with omeprazole treated 40 cases of Helicobacter pylori associated gastric ulcer. Res Tradit Chin Med. 2014;27:18–20.

[14] Pan LQ. Curative effect of Pingwei capsule combined with omeprazole on Helicobacter pylori associated gastric ulcer. Cardiovasc Dis J Integr Tradit Chin West Med. 2016;4:161.

[15] Yu AL. Clinical effect of Pingwei capsule combined with omeprazole in the treatment of Helicobacter pylori associated gastric ulcer. Stud Trace Elem Health. 2015;32:71–2.

[16] Wang QN. Effect of quadruple therapy combined with Kangfuxin solution on Helicobacter pylori positive gastric ulcer. Chin Comm Doc. 2019;35:54–6.

[17] Guo HC. Therapeutic effect of Kangfuxin solution combined with quadruple therapy on Helicobacter pylori-positive gastric ulcer. Heilongjiang J Tradit Chin Med. 2022;51:49–51.

[18] Tan DS Liang XH Liu YJ. Effect of Kangfuxin solution combined with quadruple therapy in the treatment of Helicobacter pylori-positive gastric ulcer. Chin Med Pharm. 2022;12:106–9.

[19] Zhang HY. Effect analysis of triple therapy combined with Kangfuxin solution in the treatment of gastric ulcer associated with Helicobacter pylori. Clin Res Pract. 2017;2:28–9.

[20] Yang L Yang JQ. Effect of Shugan Jieyu capsule combined with pantoprazole quadruple therapy on Helicobacter pylori associated gastric ulcer. Health Mag. 2021;10:125.

[21] Zhou M Tao Y Deng L. Clinical observation of Shugan Jieyu capsule combined quadruple therapy on the treatment of Helicobacter pylori positive gastric ulcer. Chin J Clin Gastroenterol. 2015;27:36–9.

[22] Zhang Y. The clinical efficacy of Shugan Jieyu capsule combined with quadruple therapy on Helicobacter pylori positive gastric ulcer. Chin J New Clin Med. 2018;11:464–6.

[23] Tan QJ Chen LF Li XR. Effect of Weisu granule combined with quadruple therapy on the negative conversion rate and recurrence of Helicobacter pylori in patients with Helicobacter pylori-associated gastric ulcer. J North Pharm. 2022;19:70–2.

[24] Qin YH. Application effect analysis of Weisu granule combined with quadruple therapy in the treatment of Helicobacter Pylori infected gastric ulcer. Chin J Burns. 2021;33:225–8.

[25] Yu JJ Li ZH. Application effect of Weisu granule in adjuvant treatment of Helicobacter pylori related gastric ulcer. Clin Res Pract. 2020;5:141–3.

[26] Wang XN. Effect of Qiwei Weitong capsule combined with rabeprazole quadruple therapy on Helicobacter Pylori-positive gastric ulcer. Tradit Chin West Med. 2019;19:22–4.

[27] Chen T. Clinical observation on 42 cases of Helicobacter pylori positive gastric ulcer treated by Qiwei Weitong capsule combined with quadruple therapy. Chin J Ethnobiol Ethnomed. 2019;28:95–6.

[28] Ji ZH Li FM Wu ZW. Clinical study of Beiling Weitong granule combined with improved quadruple therapy for Helicobacter Pylori-positive gastric ulcer. Pract Clin J Integr Tradit Chin West Med. 2022;22:22–5.

[29] Yu L Gong Y. Observation of the efficacy of quadruple regimen with Biling Weitong granule on Helicobacter Pylori-associated gastric ulcer. Chin J Integr Tradit Chin West Med Digestion. 2021;29:175–7.

[30] Zhang CY. Effects of Anweiyang capsule combined with esomeprazole quadruple therapy on improvement of symptoms and eradication rate of Helicobacter Pylori in patiens with Helicobacter Pylori positive gastric ulcer. Heilongjiang Med J. 2021;45:1645–1646.

[31] Chen D Zhang DF Lai YN. Effects of Anweiyang capsule combined with triple therapy on gastrointestinal hormone levels and inflammatory response in patients with gastric ulcer. Mod Med Health Res. 2022;6:86–8.

[32] Xiong YF Yuan SH Zhang QH. Clinical observation of Jinghua Weikang capsule combined with rabeprazole triple therapy in the treatment of Helicobacter pylori-related gastric ulcer. Med Forum. 2021;25:5050–2.

[33] Xu LP Yu HS. Analysis of therapeutic effect of Jinghua Weikang capsule on Helicobacter pylori positive gastric ulcer. J Shanxi Med Coll Contin Educ. 2019;29:74–6.

[34] Zhao D. Triple therapy combined Jinghua Weikang capsule or Yangweishu Helicobacter pylori eradication treatment of gastric ulcer effect was observed. Chin Foreign Med. 2015;34:131–2.

[35] Mi CY Zhao WB Yu RH. The therapeutic effect and safety of pantoprazole-based quadruple therapy combined with Jinghua Weikang capsule in patients with Helicobacter pylori infection and gastric ulcer. Guangming J Chin Med. 2022;37:2612–4.

[36] Dai W. Effects of Weifuchun tablet combined with triple therapy on Helicobacter pylori eradication rate and gastrin in patients with Helicobacter pylori-positive gastric ulcer. J Med Forum. 2018;39:151–3.

[37] Zhou YY Zhao D Y F. Clinical efficacy evaluation of Weifuchun combined with triple therapy in the treatment of Helicobacter pylori positive gastric ulcer. Contemp Med. 2018;24:129–31.

[38] Zheng CC. Study on the curative effect of Weifuchun tablet combined with quadruple therapy on Helicobacter pylori positive gastric ulcer. World Latest Med Inform. 2020;20:176–7.

[39] Zhang X Deng ML Wu LX. Triple therapy plus Weifuchun tablet for gastric ulcer infected by Helicobacter Pylori. Prog Mod Biomed. 2016;16:1937–9.

[40] Li LT Cao L. Effect of triple therapy combined with Wenweishu capsule to eradicate Helicobacter pylori in the treatment of gastric ulcer. Cardiovascu Dis J Integr Chin West Med. 2021;3:82–3.

[41] Li SS. Clinical effect of Wenweishu capsule combined with esomeprazole triple therapy on Helicobacter pylori-positive gastric ulcer patients. Chron Pathematol J. 2019;20:1223–4.

[42] Xu JY Jiang XH. Clinical study of Wenweishu capsule combined with triple therapy in the treatment of middle-Jiao deficiency cold syndrome of Helicobacter pylori positive gastric ulcer. Mod J Integr Tradit Chin West Med. 2016;25:3506–8.

[43] Zhao GL. Effect of Weidean capsule on gastric ulcer with Helicobacter pylori infection. Shenzhen J Integr Tradit Chin West Med. 2020;30:35–6.

[44] Liu M Yuan D Zhao ZQ. Clinical study on Weidan capsule combined with lansoprazole in treatment of gastric ulcer. Drugs Clinic. 2019;34:3624–8.

[45] Zheng XJ Xiao FY Yang WY. Effect of Weidean combined with quadruple therapy on curative effect and serological index of patients with gastric ulcer and Helicobacter pylori infection. Chin J Integr Tradit Chin West Med. Digestion. 2021;29:32–35.[.

[46] Wang J. Comparison of therapeutic effect of Shugan Jieyu capsule combined with pantoprazole quadruple therapy on Helicobacter pylori associated gastric ulcer. Pract Clin J Integr Tradit Chin West Med. 2020;20:109–10.

[47] Higgins JP Jackson D Barrett JK Lu G Ades AE White IR. Consistency and inconsistency in network meta-analysis: concepts and models for multi-arm studies. Res Synth Methods. 2012;3:98–110. 26062084 10.1002/jrsm.1044 PMC4433772

[48] Yoo JH Park EJ Kim SH Lee H-J. Gastroprotective effects of fermented Lotus Root against ethanol/HCl-induced gastric mucosal acute toxicity in rats. Nutrients. 2020;12:808. 32204312 10.3390/nu12030808 PMC7146638

[49] Nervi G Liatopoulou S Cavallaro LG. Does Helicobacter pylori infection eradication modify peptic ulcer prevalence? A 10 years’ endoscopical survey. World J Gastroenterol. 2006;12:2398–401. 16688832 10.3748/wjg.v12.i15.2398 PMC4088077

[50] Zeng J Xie C Zhang L. Host cell antimicrobial responses against Helicobacter pylori infection: From biological aspects to therapeutic strategies. Int J Mol Sci . 2022;23:10941. 36142852 10.3390/ijms231810941 PMC9504325

[51] Zhang WD. To explore a new way of treating Helicobacter pylori infection by combining traditional Chinese and Western medicine. Natl Med J Chin. 2012;92:2.

[52] Li Y Xu C Zhang Q Liu JY Tan RX. In vitro anti-Helicobacter pylori action of 30 Chinese herbal used to treat ulcer diseases. J Ethnopharmacol. 2005;98:329–33. 15814268 10.1016/j.jep.2005.01.020

[53] Wang JX Sun R Gao XM. Evaluation of implementation on clinical application guidelines of the Chinese patent medicine for the treatment of common diseases. Chin J Evidenced-Based Med. 2023;23:444–9.

[54] Yang L He LY Chen X. Meta-analysis of the efficacy and safety of Huanglian Wendan decoction alone or combined with Western medicine in treating insomnia caused by phlegm-heat internal disturbance. Digit Chin Med. 2022;5:340–52.

[55] Chen HX Ding HB. Efficacy and safety of Wenweishu in the treatment of gastritis and gastric ulcer. Chin Comm Doc. 2012;14:192–192.

[56] Hu FL. A multicenter study of Chinese patent medicine Wenweishu or Yangweshu in the treatment of Helicobacter pylori positive patients with chronic gastritis and gastric ulcer. Natl Med J Chin. 2010;90:75–8. 20356485

[57] Zhang LM Zhou XX Zhang QJ. Wenweishu capsule alleviates gastric mucosal damage in rats with chronic gastritis by inhibiting nuclear factor κB (NF-κB) pathway. Chin J Cell Mol Immunol. 2020;36:297–303. 32519666

[58] Yang B Wang LD Zhang J. Clinical study of combined with Pingwei capsule in the treatment of chronic gastritis caused by Helicobacter pylori with liver-Qi deficiency and damp-heat syndrome. Gansu Sci Technol. 2023;39:75–9.

[59] Wang Q Su CP Zhang HM. Anti-inflammatory mechanism of heat-clearing and detoxifying Chinese herbs. Chin J Chin Mater Med. 2018;43:3787–94. 10.19540/j.cnki.cjcmm.20180611.012 30384547

[60] Zhang ZL Wei M Yang SX. Experimental study of Pingwei capsule’s inhibition of gastric ulcer and its anti inflammatory and analgesic action. J Henan Univ Chin Med. 2003;18:26–8.

